# The linear association between vitamin B12 and diabetic retinopathy in type 2 diabetic patients: a cross-sectional study

**DOI:** 10.3389/fmed.2026.1756050

**Published:** 2026-06-22

**Authors:** Guangbi Fu, Juan Ling, Di Ling, Xinglin Chen, Wei Yang

**Affiliations:** 1 Department of Ophthalmology, Deyang People’s Hospital, Deyang, China; 2 Clinical College of Chinese Medicine, Gansu University of Chinese Medicine, Lanzhou, Gansu Province, China; 3 Department of Infection Management, Gansu Provincial Hospital, Lanzhou, Gansu Province, China; 4 School of Basic Medicine, Gansu University of Chinese Medicine, Lanzhou, Gansu Province, China; 5 Department of Geriatrics, Union Hospital, Tongji Medical College, Huazhong University of Science and Technology, Wuhan, China; 6 Department of Epidemiology and Biostatistics, Empower U, X&Y Solutions Inc., Boston, MA, United States

**Keywords:** cross-sectional study, diabetic retinopathy, generalized additive model, logistic regression, vitamin B12

## Abstract

**Objective:**

To examine the association between serum vitamin B12 levels and the prevalence of diabetic retinopathy (DR) among adults with type 2 diabetes mellitus (T2DM).

**Methods:**

This study performed a secondary analysis of a cross-sectional dataset from the Department of Endocrinology, Guangdong Provincial People’s Hospital (Dec 2017–Nov 2018). A total of 413 adults with T2DM were included, with DR adjudicated by board-certified ophthalmologists. Serum vitamin B12 was the exposure. Multivariable logistic regression assessed the vitamin B12 and DR association, alongside generalized additive models (GAMs) with smooth splines to explore nonlinearity. Prespecified subgroup and interaction analyses were conducted by sex, age, diabetes duration, blood pressure, lipid profile, and renal function.

**Results:**

After multivariable adjustment, each 100 μmol/L increase in vitamin B12 was significantly associated with a higher risk of DR (OR = 1.49, 95% CI: 1.12–1.99, *p* = 0.0067). Participants in the highest tertile of vitamin B12 (≥398 μmol/L) exhibited a substantially elevated risk of DR compared with those in the lowest tertile (OR = 11.83, 95% CI: 1.79–78.19, *p* = 0.0103). GAM indicated a linear positive association. No significant interactions were detected across prespecified subgroups (all P for interaction >0.05).

**Conclusion:**

Higher serum vitamin B12 levels were independently and linearly associated with greater DR prevalence in adults with T2DM. However, temporal ordering and causality cannot be established.

## 1 Introduction

Diabetic retinopathy (DR), as one of the most common and severe complications of diabetes, is the leading cause of blindness in diabetic patients. Its pathological mechanisms are complex, involving multiple factors such as vascular injury, inflammatory responses, and neurodegenerative changes. In recent years, research has increasingly recognized that DR is not only a pure microvascular disease but also an inflammatory and neurovascular syndrome, where neuronal damage and dysfunction often precede clinically visible microvascular lesions (1). Inflammatory responses, particularly the activation and polarization of retinal macrophages, play a key role in the pathogenesis of DR. An imbalance between pro-inflammatory (M1) and anti-inflammatory (M2) macrophages leads to the disruption of tight junctions in retinal vascular endothelium, subsequently causing increased vascular permeability and damage to the retinal barrier function (2).

Vitamin B12, as a water-soluble vitamin, is a key coenzyme for nucleic acid synthesis, fatty acid metabolism, and maintenance of nerve myelin. Its deficiency is not only associated with various neurological diseases but also closely linked to metabolic abnormalities. Clinical and basic research studies have shown that diabetic patients, especially those with retinopathy, often have vitamin B12 deficiency, and serum vitamin B12 levels are negatively correlated with the risk of DR (3, 4). Vitamin B12 deficiency is often accompanied by elevated homocysteine (Hcy) levels, and high Hcy is considered an important mediator of vascular endothelial injury and oxidative stress, potentially exacerbating the development of retinal microvascular lesions (5). Additionally, vitamin B12 exerts neuroprotective effects by regulating endoplasmic reticulum stress responses and inhibiting retinal neuronal apoptosis. In diabetic rat models, vitamin B12 supplementation can alleviate retinal ischemia and hypoxia, inhibit excessive expression of vascular endothelial growth factor (VEGF), reduce inflammation and apoptosis, thereby slowing the progression of DR (6). In recent years, nutritional intervention studies on the protective effects of vitamin B12 and related nutrients against DR have gradually increased. The combined use of multiple B vitamins (B1, B2, B6, B12) has been found to effectively reduce retinal oxidative stress and inflammatory responses, improve retinal function, and reduce the risk of DR (7, 8). Additionally, preclinical and nutritional intervention studies have suggested that vitamin B12 supplementation may modulate multiple pathogenic processes relevant to DR, including retinal ischemia/hypoxia, oxidative stress, and inflammatory/apoptotic signaling. In diabetic rat models, vitamin B12 supplementation has been reported to alleviate retinal cell apoptosis and endoplasmic reticulum stress, and to reduce dysregulated angiogenic pathways, such as VEGF and hypoxia-inducible factor 1α (HIF-1α) (6). These findings provide biological plausibility for a potential mechanistic link between B12-related metabolic pathways and DR pathophysiology, although such evidence does not establish clinical efficacy in humans.

Currently, the association between circulating vitamin B12 and DR remains inconsistent and controversial across populations and study designs. The inconsistency is evident across different study periods, populations, and exposure definitions. As early as the 1950s, conflicting findings were reported: some studies observed higher serum vitamin B12 levels in patients with DR, whereas others found increased urinary B12 excretion, resulting in contradictory conclusions (9). More recently, a large retrospective analysis in a Middle Eastern population (*n* = 1,718) reported no statistically significant association between vitamin B12 levels (≤200 vs. > 200) and retinopathy (10). In addition, meta-analytic evidence suggests that the relationship may vary by ethnicity; notably, no significant association was observed in Caucasian populations (3). Importantly, serum vitamin B12 does not exclusively reflect nutritional adequacy. Elevated circulating vitamin B12 (hypercobalaminemia) may occur secondary to underlying pathological conditions such as renal/hepatic dysfunction and chronic inflammatory states, which are also well-recognized contributors to DR risk and may distort causal inference based solely on deficiency-oriented hypotheses (11, 12). Therefore, examining serum vitamin B12 across its full observed range—including potentially elevated levels—and characterizing the functional form of the association (e.g., linearity vs. non-linearity) using generalized additive models (GAMs) may provide complementary and clinically interpretable information beyond studies focusing only on deficiency thresholds.

Therefore, this study aims to conduct a cross-sectional investigation in a specific diabetic population to evaluate the association between serum vitamin B12 levels and the prevalence and severity of DR, characterize the functional form of the relationship using generalized additive models (GAMs), explore potential influencing factors, and provide preliminary evidence for clinical screening and nutritional-risk stratification in DR.

### 1.1 Study population

This study conducted a secondary cross-sectional analysis based on the publicly available dataset from Guangdong Provincial People’s Hospital (Dryad, doi:10.5061/dryad.6kg1sd7) (13). This database encompasses healthcare data concerning 503 patients with type 2 diabetes from southern China, ensuring the anonymity of individual participants by excluding identifiable information. Data collection occurred between December 2017 and November 2018. After excluding patients missing data in DR, our study ultimately involved 413 individuals. Source data were gathered from December 2017 to November 2018 via a cross-sectional survey administered in the endocrinology department of Guangdong Provincial People’s Hospital.

The inclusion criteria were as follows: (1) individuals diagnosed with type 2 diabetes based on the criteria established by the World Health Organization (14); (2) color retinal photographs taken at 35 degrees using 7-standard fields from the Early Treatment Diabetic Retinopathy Study (ETDRS) (15); (3) Patients visited in the endocrinology department of Guangdong Provincial People’s Hospital from December 2017 to November 2018.

The exclusion criteria comprised: (1) the elimination of any other ocular conditions that could influence ocular circulation (such as glaucoma, endophthalmitis, retinal vascular occlusion, age-related macular degeneration, refractive errors exceeding 3 degrees, or ocular trauma); (2) the presence of significant systemic illnesses (including myocardial infarction, cerebral infarction, or connective tissue disorders); (3) a prior history of intravitreal injections or dialysis; (4) women who are pregnant or menstruating. Since patient data was collected retrospectively and has been anonymized and de-identified, obtaining signed informed consent was not necessary.

A diagnosis was made by any of the following: (1) fasting plasma glucose ≥7.0 mmol/L (126 mg/dL); (2) 2-h plasma glucose ≥11.1 mmol/L (200 mg/dL) during a 75-g oral glucose tolerance test; (3)HbA1c ≥ 6.5% (48 mmol/mol); or (4) in a patient with classic symptoms of hyperglycemia or hyperglycemic crisis, a random plasma glucose ≥11.1 mmol/L (200 mg/dL). In the absence of unequivocal hyperglycemia, results for criteria (1)–(3) required confirmation by repeat testing on a separate day (16).

### 1.2 Ethics statement

The anonymized dataset analyzed in this secondary study was derived from Zhuang et al. (13). This study was approved by the Research Ethics Committee of Guangdong Provincial People’s Hospital (registration number: gdrec2016232A). All participants provided written informed consent that covered study participation and the publication of de-identified clinical information. Because the present work involved secondary analysis of a publicly available, de-identified dataset and entailed no new recruitment or interventions, no additional ethics approval was required.

### 1.3 Variables

All medical data were obtained from the patient’s medical records. The demographic and physiological information encompassed sex, age, duration of diabetes mellitus (DM), height, weight, and blood pressure. The exposure variable was serum vitamin B12 concentration, measured using an electrochemiluminescence immunoassay (ECLIA), with results expressed in μmol/L. Serum vitamin B12 concentrations were reported in μmol/L in the original dataset from the BMJ Open study by Zhuang et al. (13), which we used for this secondary analysis; therefore, we retained the same unit for consistency. For clinical context, the conventional reference interval for serum vitamin B12 is commonly reported as 148–664 pmol/L (with deficiency often defined as <148 pmol/L), although laboratory- and assay-specific cut-offs may vary. Vitamin B12 levels were treated as a continuous variable, and our statistical analyses were based on the original dataset values as reported.

### 1.4 Covariates

The covariates incorporated in this investigation encompassed: gender, age, duration of diabetes mellitus, glycated hemoglobin (HbA1c), blood urea nitrogen (BUN), high-density lipoprotein (HDL), low-density lipoprotein (LDL), triglycerides (TRIG), total cholesterol (CHOL), total protein (TP), diastolic blood pressure (DBP), pulse pressure (PP), estimated glomerular filtration rate (eGFR), and the stage of chronic kidney disease (CKD stage).

### 1.5 DR confirmation

Confirmation of DR was meticulously performed by a team of board-certified ophthalmologists, who conducted thorough and comprehensive ocular examinations to ensure accurate diagnosis. The existence or non-existence of DR constituted the principal outcome measure in this investigation. In alignment with the International Clinical Diabetic Retinopathy and Diabetic Macular Edema Disease Severity Scale (17), the identification of DR was documented as 1 (DR), whereas its absence was denoted as 0 (NDR).

### 1.6 Statistical analysis

Continuous variables were presented as mean ± standard deviation or median (interquartile range) according to their distributional characteristics, while categorical variables were expressed as frequencies (percentages). For between-group comparisons, normally distributed continuous variables were analyzed using the independent-samples *t*-test, non-normally distributed continuous variables were assessed with the Mann–Whitney U test, and categorical variables were compared using the chi-square test.

The relationship between vitamin B12 and DR was evaluated with multivariable logistic regression combined with smooth curve fitting, adjusting for relevant clinical covariates. Because the outcome (DR: yes/no) is binary, we selected multivariable logistic regression to estimate the adjusted odds of DR. To avoid relying on a purely linear assumption, we used generalized additive models (GAMs) with smooth splines to flexibly assess the functional form of the association between serum vitamin B12 and DR prevalence. The smooth-curve approach allowed us to evaluate whether the relationship was approximately linear or potentially non-linear. An unadjusted model (Model I) was first constructed, followed by sequential adjustment for potential confounders: Model II was adjusted for sex, age. Model III further adjusted for sex, age, SBP, DBP, HbA1c, Blood Urea Nitrogen, NEFA, HDL, LDL, TRIG, CHOL, Lpa, APOA, APOB, ALT, AST, AchE, ALB, TP and D-dimer. Vitamin B12 was included in the models both as a continuous variable (per unit increase) and as a categorical variable based on tertiles (with the lowest tertile as the reference). Results were reported as odds ratios (ORs) with corresponding 95% confidence intervals (CIs).

Subgroup analyses were conducted according to sex, age, diabetes duration, systolic blood pressure, diastolic blood pressure, HbA1c, BUN, HDL, LDL, CHOL, TP, and eGFR. Heterogeneity across subgroups was evaluated by incorporating interaction terms between vitamin B12 and each subgroup variable into the logistic regression models, with *p* values for interaction reported. All analyses were performed with EmpowerStats, version 4.1 (X&Y Solutions, Inc., Boston, MA; www.empowerstats.net), and the R environment, version 4.2.0 (The R Foundation; http://www.r-project.org).

## 2 Result

A total of 413 patients with type 2 diabetes were included in this study, of whom 253 had no diabetic retinopathy (non-diabetic retinopathy, NDR) and 160 had confirmed DR. Baseline characteristics for the NDR and DR groups are summarised in Table 1.

**Table 1:** Baseline characteristics of the study participants between two groups (*n* = 413).

Characteristics	NDR (*n* = 253)	DR (*n* = 160)	*p* value
General			
Male, *n* (%)	157 (62.06%)	76 (47.50%)	0.004
Female, *n* (%)	96 (37.94%)	84 (52.50%)	
Age (years)	58.08 ± 13.74	60.34 ± 12.84	0.095
DM duration (years)	6.00 (1.00–12.00)	10.50 (8.00–18.00)	<0.001
SBP (mmHg)	135.52 ± 19.19	143.61 ± 23.18	<0.001
DBP (mmHg)	80.19 ± 11.66	80.12 ± 12.46	0.956
HbA1c (%)	9.54 ± 2.65	9.74 ± 2.30	0.453
Renal parameters			
Blood urea nitrogen (mg/dL)	5.39 (4.32–6.73)	6.13 (4.88–8.76)	0.022
eGFR (mL/min/1.73 m^2^)	89.84 ± 27.95	75.13 ± 39.50	<0.001
UACR (mg/g)	5.85 (3.30–17.92)	54.52 (10.04–521.78)	<0.001
UPCR (mg/g)	92.30 (63.38–162.20)	239.19 (113.40–1076.15)	<0.001
Ucr (μmol/L)	92.49 (75.88–103.46)	80.30 (48.52–98.50)	0.002
Blood lipid			
NEFA (mmol/L)	0.40 ± 0.22	0.33 ± 0.20	0.001
HDL (mmol/L)	1.00 ± 0.34	1.06 ± 0.32	0.065
LDL (mmol/L)	3.10 ± 0.93	3.36 ± 1.13	0.013
TRIG (mmol/L)	1.52 (1.10–2.26)	1.50 (0.97–2.35)	0.512
Total cholesterol (mmol/L)	4.85 ± 1.45	5.26 ± 1.88	0.013
Lp(a) (mg/L)	100.00 (48.00–217.00)	122.50 (73.75–253.25)	0.052
ApoA (g/L)	1.04 ± 0.41	1.10 ± 0.37	0.084
ApoB (g/L)	0.84 ± 0.37	0.92 ± 0.38	0.033
Others			
ALT (U/L)	20.00 (15.00–29.00)	13.00 (12.00–23.00)	0.007
AST (U/L)	20.00 (16.00–25.00)	17.00 (14.00–22.00)	0.02
AChE (U/L)	8462.63 ± 2000.92	8235.36 ± 2244.07	0.284
Albumin (g/L)	37.95 ± 4.60	35.74 ± 5.75	<0.001
Total protein (g/L)	65.36 ± 7.01	64.57 ± 6.44	0.25
D-dimer (μg/L)	330.00 (270.00–500.00)	435.00 (290.00–740.00)	0.001
Vitamin B12 (μmol/L)	287.00 (171.00–435.00)	327.50 (206.50–549.25)	0.004

DM, diabetes mellitus; SBP, systolic blood pressure; DBP, diastolic blood pressure; HbA1c, glycated hemoglobin A1c; eGFR, estimated glomerular filtration rate; UACR, urinary albumin-to-creatinine ratio; UPCR, urinary protein-to-creatinine ratio; Ucr, urinary creatinine; NEFA, non-esterified fatty acids; HDL, high-density lipoprotein cholesterol; LDL, low-density lipoprotein cholesterol; Lp(a), lipoprotein(a); ApoA, apolipoprotein A-I; ApoB, apolipoprotein B; ALT, alanine aminotransferase; AST, aspartate aminotransferase; AChE, acetylcholinesterase.

Table 2 summarizes the association between vitamin B12 and DR. In the primary effect analysis, each 100 μmol/L increase in vitamin B12 was associated with an elevated prevalence of DR (Table 2). In the unadjusted model (Model 1), the odds ratio (OR) was 1.11 (95% CI: 1.03–1.19, *p* = 0.0049). After adjustment for age and sex (Model 2), the OR was 1.09 (95% CI, 1.02–1.17, *p* = 0.0146). Further adjustment for all covariates—including blood pressure, glycemic control, renal function, lipid profile, liver enzymes, inflammatory markers, and coagulation parameters (Model 3)—strengthened the association substantially, with the OR increasing to 1.49 (95% CI, 1.12–1.99, *p* = 0.0067). Stratification by tertiles of vitamin B12 revealed that participants in the highest tertile (≥398 μmol/L) exhibited a significantly increased risk of DR in the fully adjusted model (OR = 11.83, 95% CI: 1.79–78.19, *p* = 0.0103).

**Table 2:** The association between vitamin B12 and DR.

Vitamin B12 (per 100 μmol/L)	Model 1	Model 2	Model 3
	[OR (95% CI), *p*]	[OR (95% CI), *p*]	[OR (95% CI), *p*]
Overall	1.11 (1.03, 1.19) 0.0049	1.09 (1.02, 1.17) 0.0146	1.49 (1.12, 1.99) 0.0067
Vitamin B12 tertile			
Low (0–231 μmol/L)	1	1	1
Middle (233–397 μmol/L)	1.19 (0.72, 1.95) 0.4980	1.27 (0.77, 2.11) 0.3499	1.32 (0.26, 6.76) 0.7387
High (398-1107 μmol/L)	1.84 (1.13, 3.00) 0.0144	1.77 (1.08, 2.90) 0.0240	11.83 (1.79, 78.19) 0.0103

Model 1: no covariates were adjusted; Model 2: sex and age were adjusted; Model 3: sex, age, SBP, DBP, HbA1c, Blood Urea Nitrogen, NEFA, HDL, LDL, TRIG, CHOL, Lpa, APOA, APOB, ALT, AST, AchE, ALB, TP and D-dimer were adjusted.

Subgroup analyses and interaction tests were performed to further examine the association between the vitamin B12 and DR and evaluate potential factors that may influence this correlation (Table 3). Subgroup analyses were conducted by sex (male or female), age tertiles (19.00–35.00, 54.00–64.00, and 65.00–92.00 years), diabetes duration tertiles (1.00–4.00, 5.00–11.00, and 12.00–59.00 years), and tertiles of systolic blood pressure (SBP; mm Hg), diastolic blood pressure (DBP; mm Hg), HbA1c (%), blood urea nitrogen (mg/dl), HDL (mmol/L), LDL (mmol/L), total cholesterol (CHOL; mmol/L), total protein (TP; g/L), and estimated glomerular filtration rate (eGFR; mL/min/1.73 m^2^), each defined from the sample distribution. No significant interactions were detected across these subgroups (all P for interaction > 0.05).

**Table 3:** Effect size of vitamin B12 on DR in prespecified and exploratory subgroups.

Subgroups	Without DR	With DR	OR (95% CI)	*p* value	P for interaction
Sex					0.736
Male	157 (62.1%)	76 (47.5%)	1.01 (1.00, 1.03)	0.0458	
Female	96 (37.9%)	84 (52.5%)	1.01 (0.99, 1.02)	0.3016	
Age tertile					0.841
Low (19.00–35.00)	92 (36.4%)	45 (28.1%)	1.01 (0.98, 1.04)	0.4461	
Middle (54.00–64.00)	76 (30.0%)	56 (35.0%)	1.01 (0.99, 1.03)	0.4119	
High (65.00–92.00)	85 (33.6%)	59 (36.9%)	1.01 (1.00, 1.03)	0.0276	
DM duration (years)					0.455
Low (1.00–4.00)	109 (43.1%)	22 (13.8%)	1.01 (0.98, 1.04)	0.6427	
Middle (5.00–11.00)	77 (30.4%)	60 (37.5%)	1.01 (0.99, 1.02)	0.3968	
High (12.00–59.00)	67 (26.5%)	78 (48.8%)	1.02 (1.01, 1.03)	0.0064	
SBP (mm Hg) tertile					
Low (92.00–127.00)	88 (34.8%)	48 (30.0%)	1.02 (0.99, 1.04)	0.1267	0.616
Middle (128.00–145.00)	94 (37.2%)	41 (25.6%)	1.01 (1.00, 1.03)	0.0849	
High (146.00–204.00)	71 (28.1%)	71 (44.4%)	1.01 (0.99, 1.02)	0.2095	
DBP (mm Hg) tertile					0.619
Low (50.00–74.00)	79 (31.2%)	52 (32.5%)	1.01 (0.99, 1.02)	0.4039	
Middle (75.00–84.00)	87 (34.4%)	53 (33.1%)	1.02 (1.00, 1.04)	0.0101	
High (85.00–127.00)	87 (34.4%)	55 (34.4%)	1.01 (0.99, 1.03)	0.2436	
HbA1c (%) tertile					
Low (0.00–8.20)	85 (33.6%)	49 (30.6%)	1.01 (1.00, 1.03)	0.0837	0.851
Middle (8.30–10.50)	86 (34.0%)	54 (33.8%)	1.02 (1.00, 1.04)	0.0134	
High (10.60–16.30)	82 (32.4%)	57 (35.6%)	1.01 (0.99, 1.03)	0.3327	
Blood urea nitrogen (mg/dl) tertile					0.331
Low (2.06–4.80)	97 (38.3%)	40 (25.0%)	1.02 (1.00, 1.04)	0.0164	
Middle (4.87–6.59)	86 (34.0%)	51 (31.9%)	1.01 (0.99, 1.03)	0.2681	
High (6.60–353.00)	70 (27.7%)	69 (43.1%)	1.01 (0.99, 1.02)	0.303	
HDL (mmol/L) tertile					0.421
Low (0.00–0.87)	89 (35.2%)	47 (29.4%)	1.03 (1.01, 1.05)	0.0026	
Middle (0.88–1.07)	86 (34.0%)	52 (32.5%)	1.00 (0.98, 1.01)	0.7629	
High (1.08–3.43)	78 (30.8%)	61 (38.1%)	1.02 (1.00, 1.04)	0.0264	
LDL (mmol/L) tertile					0.369
Low (0.00–2.72)	88 (34.8%)	49 (30.6%)	1.01 (0.99, 1.02)	0.3166	
Middle (2.73–3.58)	96 (37.9%)	42 (26.2%)	1.01 (0.99, 1.04)	0.1544	
High (3.59–6.59)	69 (27.3%)	69 (43.1%)	1.02 (1.00, 1.03)	0.0519	
CHOL (mmol/L) tertile					0.286
Low (0.00–4.26)	84 (33.2%)	51 (31.9%)	1.00 (0.99, 1.02)	0.5061	
Middle (4.30–5.48)	96 (37.9%)	39 (24.4%)	1.02 (1.00, 1.04)	0.0891	
High (5.55–13.70)	73 (28.9%)	70 (43.8%)	1.02 (1.00, 1.03)	0.0345	
TP (g/L) tertile					0.207
Low (0.00–63.40)	79 (31.2%)	59 (36.9%)	1.02 (1.00, 1.04)	0.0556	
Middle (63.50–67.10)	80 (31.6%)	55 (34.4%)	1.01 (1.00, 1.03)	0.1615	
High (67.20–85.00)	94 (37.2%)	46 (28.7%)	1.01 (0.99, 1.02)	0.3054	
eGFR (mL/min/1.73m^2^) tertile					0.115
Low (7.24–75.92)	66 (26.1%)	72 (45.0%)	1.01 (1.00, 1.02)	0.1098	
Middle (76.25–98.18)	91 (36.0%)	46 (28.7%)	1.00 (0.99, 1.02)	0.6257	
High (98.23–374.89)	96 (37.9%)	42 (26.2%)	1.03 (1.01, 1.05)	0.0113	

Sex, age, DM duration, SBP, DBP, HbA1c, HDL, LDL, CHOL, TP, Blood Urea Nitrogen and eGFR were adjusted.

In each stratified analysis, the model is not adjusted for the stratification variable.

**Figure 1:**
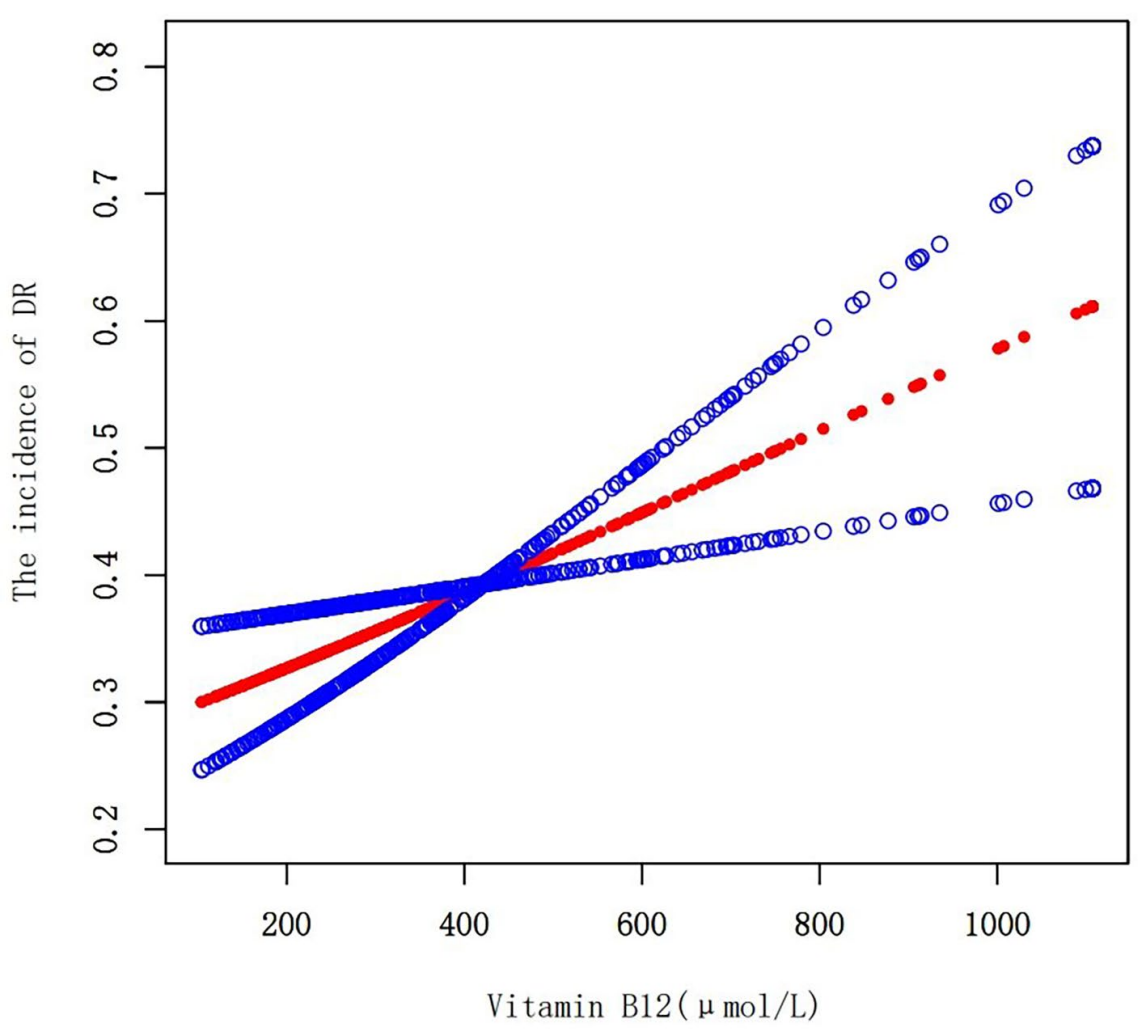
The association between vitamin B12 and DR.

**Figure 2:**
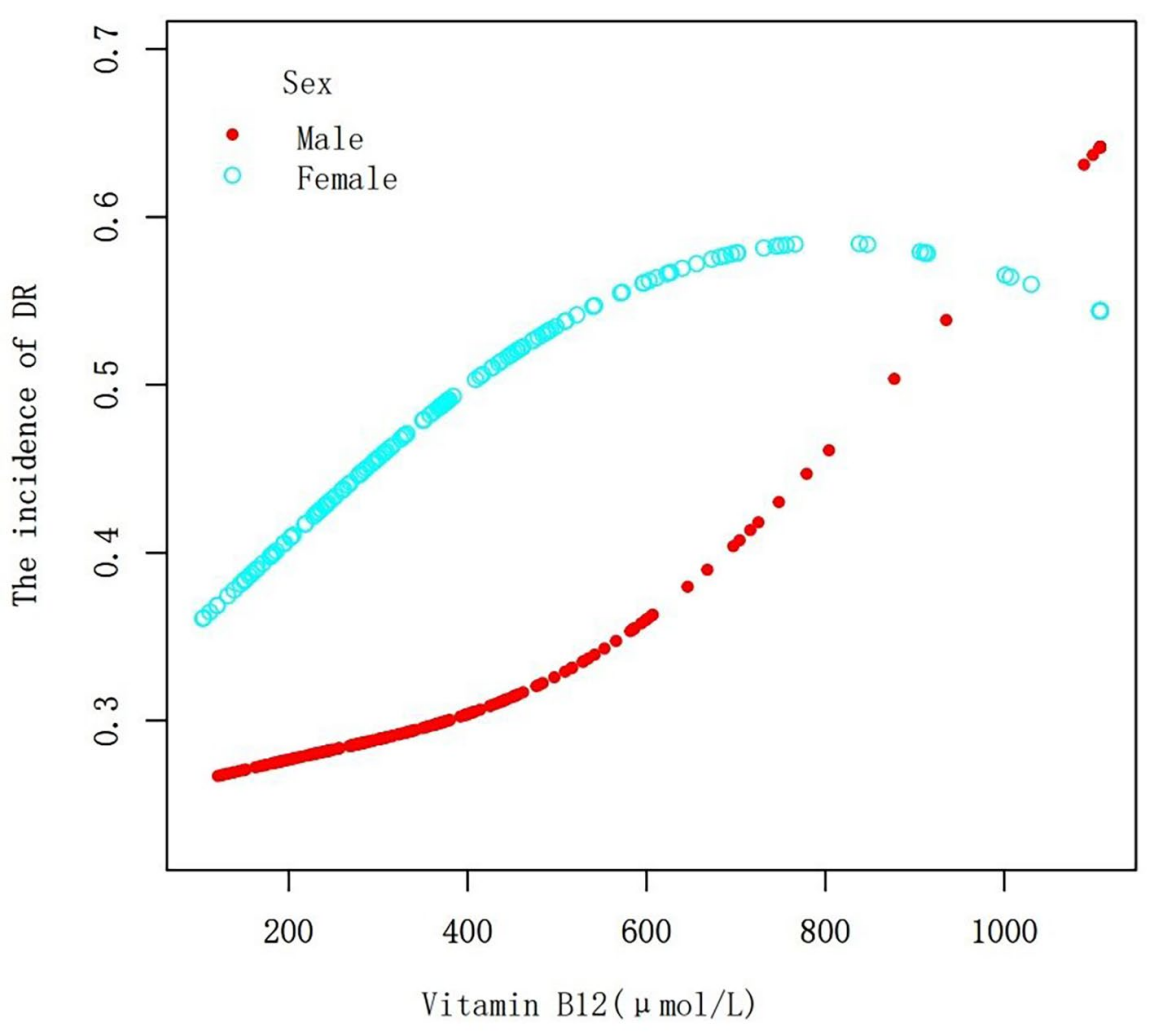
Subgroup analysis stratified by sex.

**Figure 3:**
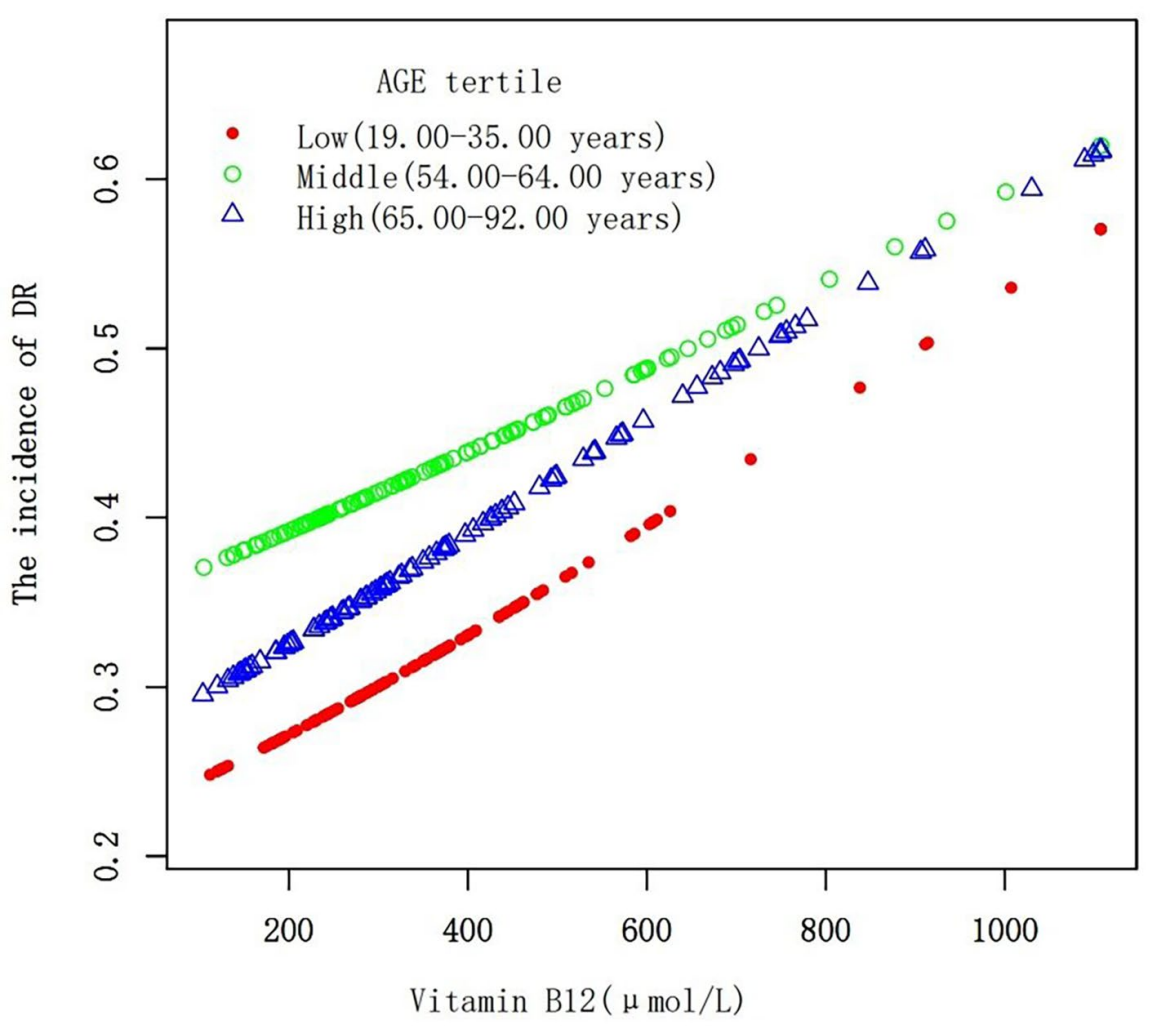
Subgroup analysis stratified by age.

**Figure 4:**
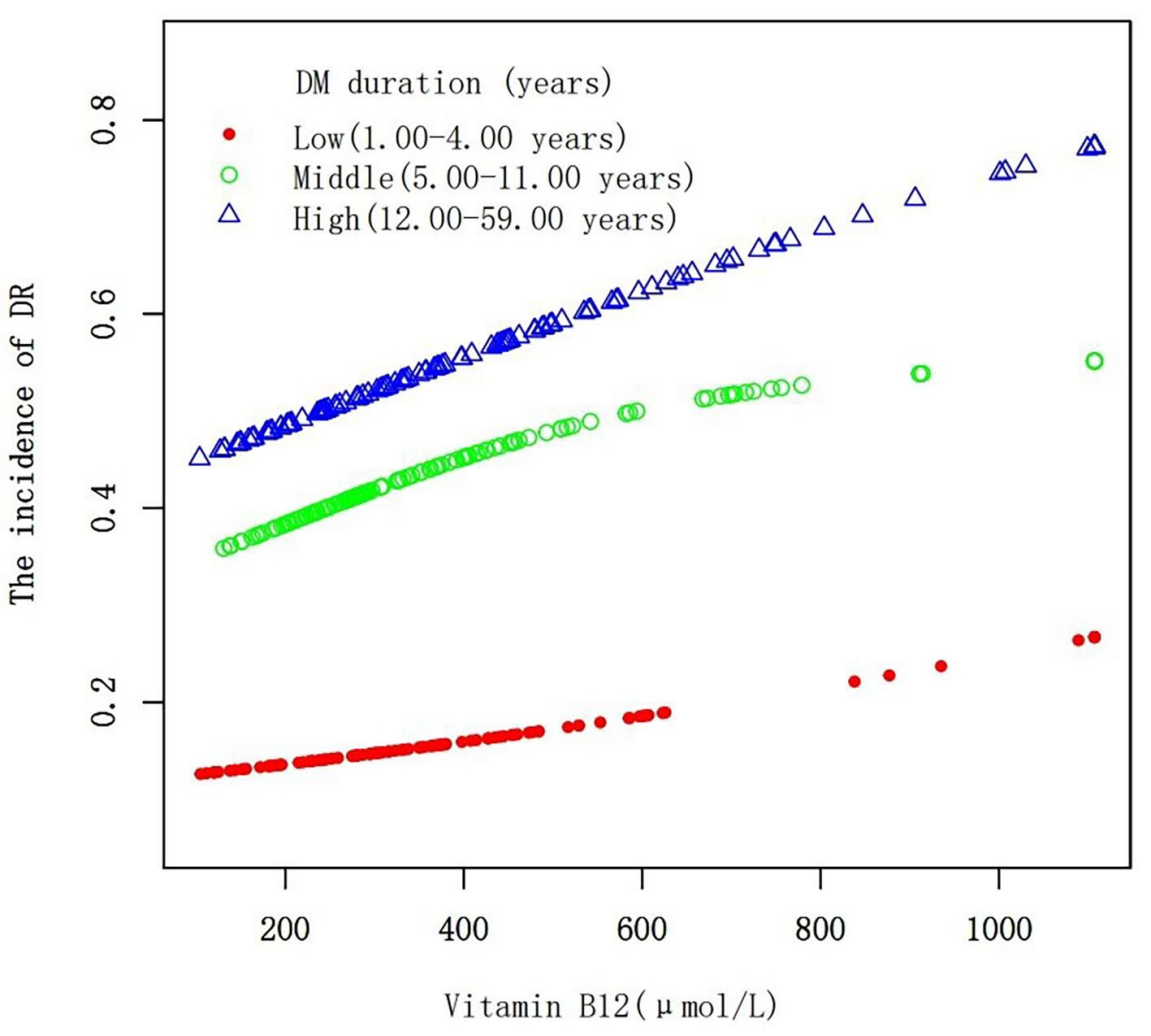
Subgroup analysis stratified by DM duration.

Note: We employed generalized additive models (GAMs) to a generalized additive model (GAM) was used to test the non-linear relationship between DR and vitamin B12 and to create the smooth curve plots and the *p*-value for non-linearity in Figures 1–4 represents the value of the likelihood ratio test for threshold effects Figure 1. The association between vitamin B12 and DR. Red line represents the smooth curve. Blue bands represent the 95% confidence interval. Sex, age, BMI, cholesterol, triglycerides, LDL, HDL, creatinine, eGFR, ischaemic heart disease, ACEI and/or ARB use, *β*-blocker use, UACR>30 mg/g, Calcium channel blocker use and ABI were adjusted Figure 2. Subgroup analysis stratified by age. Sex, BMI, cholesterol, triglycerides, LDL, HDL, Creatinine, eGFR, Ischaemic Heart Disease, ACEI and/or ARB use, β-blocker use, UACR>30 mg/g, calcium channel blocker use, and ABI were adjusted Figure 3. Subgroup analysis stratified by BMI. Sex, age, cholesterol, triglycerides, LDL, HDL, Creatinine, eGFR, ischaemic heart disease, ACEI and/or ARB use, β-blocker use, UACR>30 mg/g, Calcium channel blocker use and ABI were adjusted Figure 4. Subgroup analysis stratified by Sex. Age, BMI, cholesterol, triglycerides, LDL, HDL, Creatinine, eGFR, Ischaemic Heart Disease, ACEI and/or ARB use, β-blocker use, UACR>30 mg/g, calcium channel blocker use, and ABI were adjusted.

Smooth-spline fitting demonstrated a clear, approximately linear positive association between vitamin B12 and the prevalence of DR: Figure 1 demonstrates a linear positive association between serum vitamin B12 and DR. DR probability increased steadily with rising B12 concentrations, without evidence of a threshold. Sex-stratified analysis (Figure 2) showed parallel positive slopes for males and females, with no significant between-sex difference. Age-stratified analysis (Figure 3) revealed comparable regression slopes across the low (19–35 years), middle (54–64 years) and high (65–92 years) age tertiles, indicating that age did not materially modify the B12-DR relationship. Likewise, DM-duration stratification (Figure 4) exhibited consistent positive linear trends in short (1–4 years), intermediate (5–11 years) and long (12–59 years) duration subgroups, with a slight steepening of the slope as duration increased, suggesting that longer diabetes duration may amplify the strength of the B12-DR association. Overall, vitamin B12 is linearly and positively associated with DR prevalence, and this association is homogeneous across sex, age and DM-duration strata.

## 3 Discussion

This study is a clinical cross-sectional investigation aimed at exploring the association between serum vitamin B12 levels and DR. After comprehensive adjustment for potential confounders—including sex, age, systolic and diastolic blood pressure, glycated hemoglobin (HbA1c), blood urea nitrogen, non-esterified fatty acids, lipid profile, liver function parameters, albumin, total protein, and D-dimer—the results demonstrated that each 100 μmol/L increase in serum vitamin B12 was associated with a 49% higher risk of DR (OR = 1.49, 95% CI: 1.12–1.99, *p* = 0.0067). Furthermore, compared with the lowest tertile (<231 μmol/L), individuals in the highest tertile (≥398 μmol/L) exhibited an approximately 11-fold increased risk of DR (OR = 11.83, 95% CI: 1.79–78.19, *p* = 0.0103). Notably, the wide confidence interval indicates substantial imprecision, which may reflect limited information within the highest tertile and potential sparse-data effects. Importantly, consistent evidence was also observed when B12 was modeled as a continuous exposure (OR = 1.49 per 100 μmol/L, 95% CI: 1.12–1.99), and the GAM smooth-spline analysis suggested an approximately linear dose–response relationship without evidence of nonlinearity. Therefore, the elevated tertile estimate should be interpreted cautiously, while the overall positive association appears supported across alternative specifications. These findings suggest a significant positive association between elevated serum vitamin B12 levels and DR prevalence among individuals with diabetes in southern China.

Our results contrast markedly with the majority of prior literature on the relationship between vitamin B12 and DR. A meta-analysis by Yang et al. (3) of 15 studies demonstrated that, among patients with type 2 diabetes, those with DR exhibited significantly lower serum vitamin B12 levels than those without DR (weighted mean difference [WMD] = −68.91 μmol/L), with this inverse association being particularly pronounced in East and South Asian populations. Similarly, a case–control study by Debnath et al. (4) reported a significant association between vitamin B12 deficiency and DR, proposing that this link may be mediated through hyperhomocysteinemia-induced retinal microvascular damage. Additionally, a cross-sectional analysis of NHANES data by Zhou et al. (18) found that levels of multiple B vitamins, including B12, progressively declined with increasing DR severity. In contrast, our study observed a positive association between elevated vitamin B12 levels and higher DR prevalence. This apparent discrepancy may be explained by the pathophysiological nature of hypercobalaminemia. As noted by Andrès et al. (11) and Serraj et al. (12), elevated serum vitamin B12 levels often do not reflect nutritional adequacy but rather serve as a biomarker of underlying severe conditions, such as hepatic or renal dysfunction, hematologic malignancies, or chronic inflammatory states. In our cohort, DR patients exhibited significantly lower estimated glomerular filtration rate (eGFR), higher urinary albumin-to-creatinine ratio (UACR), and reduced total protein levels—indicative of more advanced renal impairment. Given that renal dysfunction is a recognized contributor to hypercobalaminemia (19), elevated B12 levels in patients with DR may reflect underlying disease severity—particularly renal impairment—rather than indicating a direct causal role in DR. Importantly, however, not all DR patients necessarily have clinically significant kidney dysfunction. In our cohort, DR participants showed worse renal parameters (lower eGFR and higher UACR), consistent with this possibility. To further evaluate whether the B12–DR association differed by renal function, we performed renal-function stratified analyses using eGFR tertiles (Table 3). No significant interaction was observed across eGFR tertiles (P for interaction = 0.115), suggesting that the association was broadly consistent across renal function strata. Nevertheless, because the study is cross-sectional, reverse causality and shared underlying pathophysiology cannot be fully excluded. Elevated serum B12 (hypercobalaminemia) can occur in individuals with reduced renal clearance and/or systemic inflammation, both common in advanced diabetes. Therefore, our findings should be interpreted as supporting an association in which elevated B12 may serve as a biochemical marker of disease severity, pending confirmation from prospective mechanistic studies.

The clinical implications of our findings underscore the need for cautious interpretation of elevated serum vitamin B12 levels in patients with diabetes. Although vitamin B12 is commonly regarded as a neuroprotective and antioxidant micronutrient (7, 8), its elevated serum concentration in specific clinical contexts may signal underlying pathology rather than nutritional benefit. Our results emphasize that vitamin B12 levels should not be evaluated in isolation when assessing DR risk; instead, they must be interpreted in conjunction with renal function, inflammatory status, and other metabolic parameters. In contrast to previous studies focusing on B12 deficiency and microvascular complications (20), our work uniquely highlights the potential warning value of high B12 levels in individuals with DR, offering a novel perspective for clinical risk stratification. From a public health standpoint, this finding suggests that diabetes management should pay closer attention to “abnormally high” rather than solely “low” vitamin B12 concentrations. Future research should employ prospective cohort designs and incorporate functional biomarkers—such as holotranscobalamin (holoTC), methylmalonic acid (MMA), and homocysteine—to distinguish true B12 excess from secondary hypercobalaminemia and to elucidate causal relationships with DR progression.

This study possesses several methodological strengths. First, it was conducted in the Department of Endocrinology at Guangdong Provincial People’s Hospital and included 413 patients with diabetes—a sample size representative of similar cross-sectional studies. All participants underwent standardized assessment for DR status, enhancing phenotypic accuracy. Second, we comprehensively adjusted for a wide range of potential confounders using available laboratory and clinical variables, including demographic variables (sex, age), diabetes-related metrics (duration, HbA1c), renal parameters (eGFR, blood urea nitrogen [BUN], UACR), lipid profiles (HDL, LDL, triglycerides, total cholesterol), and blood pressure. However, residual confounding may still exist because several clinically relevant factors (e.g., vitamin B12 supplementation, metformin use, clinical liver disease, and systemic inflammatory markers such as CRP) were not available for adjustment in this dataset. Third, our statistical approach was rigorous: in addition to continuous variable analysis, we employed tertile-based stratification to capture potential non-linear relationships and conducted subgroup analyses (by sex, age, and diabetes duration) to assess result robustness. All interaction *p* values for subgroup analyses exceeded 0.05, indicating consistent main effects across subpopulations. Furthermore, the study adhered strictly to the STROCSS 2024 guidelines (21) for reporting cross-sectional studies, enhancing transparency and reproducibility.

Nevertheless, several limitations warrant consideration. First, because this is a cross-sectional observational study, our results should be interpreted as association only, and no causal or temporal relationship between vitamin B12 levels and DR can be established. Thus, it remains unclear whether elevated B12 is a cause, a consequence, or merely a concomitant marker of shared pathophysiological processes. Second, because the study was conducted in a single tertiary hospital in a Chinese population, our findings may not be generalizable to other regions or global populations. Third, several clinically important variables were not available for adjustment in this dataset, including vitamin B12 supplementation, metformin use, liver disease, and systemic inflammatory markers such as C-reactive protein (CRP). Consequently, residual confounding cannot be fully ruled out. The association magnitude and functional form may vary across populations due to differences in baseline risk, the prevalence and severity of renal impairment, clinical practices regarding vitamin B12 measurement/supplementation, and ethnic/health-system factors. Fourth, because this study used a publicly available dataset from Dryad for secondary analysis, our study was further constrained by variable availability and we had no control over the original data collection and laboratory measurement procedures, as well as the handling of missing data in the source study. Accordingly, large-scale, multi-center, multi-ethnic prospective studies—and, where feasible, interventional research—are needed to validate and extend our findings and to clarify the temporal relationship between vitamin B12 and DR. In addition, a formal mediation analysis (e.g., through renal impairment or inflammatory pathways) is not feasible in this dataset because mechanistic mediators and time-ordered measurements are limited.

## 4 Conclusion

In this cross-sectional study, we observed a significant positive association between serum vitamin B12 levels and the prevalence of DR in patients with type 2 diabetes. Participants with higher vitamin B12 concentrations exhibited an increased risk of DR, independent of traditional risk factors such as age, sex, glycemic control, and renal function. However, due to the cross-sectional design, temporal ordering and causality cannot be determined. These findings challenge the conventional view of vitamin B12 as a protective factor and suggest that elevated B12 levels may serve as a marker of underlying metabolic or renal dysfunction rather than a direct contributor to DR. Our results highlight the importance of interpreting vitamin B12 levels within the broader clinical context, particularly in patients with diabetes. Future prospective and mechanistic studies are needed to determine whether hypercobalaminemia is a surrogate marker of disease severity or whether an independent etiologic role exists in the development or progression of DR—questions that cannot be answered by the present cross-sectional design.

## Data Availability

The datasets presented in this study can be found in online repositories. The names of the repository/repositories and accession number(s) can be found in the article/Supplementary material.

## References

[1] Sinclair SH Schwartz SS. Diabetic retinopathy-an underdiagnosed and undertreated inflammatory, neuro-vascular complication of diabetes. Front Endocrinol (Lausanne). (2019) 10:843. doi: 10.3389/fendo.2019.00843, 31920963 PMC6923675

[2] Lee SJ Noh SE Jo DH Cho CS Park KS Kim JH. IL-10-induced modulation of macrophage polarization suppresses outer-blood-retinal barrier disruption in the streptozotocin-induced early diabetic retinopathy mouse model. FASEB J. (2024) 38:e23638. doi: 10.1096/fj.202400053R, 38713098

[3] Yang X Hu R Zhu Y Wang Z Hou Y Su K . Meta-analysis of serum vitamin B12 levels and diabetic retinopathy in type 2 diabetes. Arch Med Res. (2023) 54:64–73. doi: 10.1016/j.arcmed.2022.12.006, 36549948

[4] Debnath PR Debnath DK Haque AF Mohammuddunnobi M Bhowmik NC. Role of vitamin B12 deficiency and hyperhomocysteinemia in diabetic retinopathy. Mymensingh Med J. (2023) 32:459–62. 37002758

[5] Tomić M Vrabec R Ljubić S Bulum T Rahelić D. Plasma homocysteine is associated with nonproliferative retinopathy in patients with type 2 diabetes without renal disease. Diabetes Metab Syndr. (2022) 16:102355. doi: 10.1016/j.dsx.2021.102355, 34920196

[6] Reddy SS Prabhakar YK Kumar CU Reddy PY Reddy GB. Effect of vitamin B12 supplementation on retinal lesions in diabetic rats. Mol Vis. (2020) 26:311–25. 32355441 PMC7190579

[7] Shi C Wang P Airen S Brown C Liu Z Townsend JH . Nutritional and medical food therapies for diabetic retinopathy. Eye Vis (Lond). (2020) 7:33. doi: 10.1186/s40662-020-00199-y, 32582807 PMC7310218

[8] Ruamviboonsuk V Grzybowski A. The roles of vitamins in diabetic retinopathy: a narrative review. J Clin Med. (2022) 11:6490. doi: 10.3390/jcm11216490, 36362717 PMC9656452

[9] Heaton JM. Vitamin B12 and the eye. Proc Nutr Soc. (1960) 19:100–5. doi: 10.1079/PNS19600024, 14400448

[10] Swidan AK Ahmed MASA. Should we follow the guidelines on vitamin B12 deficiency and diabetes? A retrospective analysis of data from middle eastern population. Alexandria J Med. (2023) 59:36–41. doi: 10.1080/20905068.2023.2209410

[11] Andrès E Serraj K Zhu J Vermorken AJ. The pathophysiology of elevated vitamin B12 in clinical practice. QJM. (2013) 106:505–15. doi: 10.1093/qjmed/hct051, 23447660

[12] Serraj K Mecili M Housni I Andrès E. Hypervitaminemia B12 (high level of cobalamin): physiopathology, role and interest in clinical practice. Presse Med. (2011) 40:1120–7. doi: 10.1016/j.lpm.2011.08.010, 22023830

[13] Zhuang X Cao D Yang D Zeng Y Yu H Wang J . Association of diabetic retinopathy and diabetic macular oedema with renal function in southern Chinese patients with type 2 diabetes mellitus: a single-Centre observational study. BMJ Open. (2019) 9:e031194. doi: 10.1136/bmjopen-2019-031194, 31494622 PMC6731866

[14] Alberti KG Zimmet PZ. Definition, diagnosis and classification of diabetes mellitus and its complications. Part 1: diagnosis and classification of diabetes mellitus provisional report of a WHO consultation. Diabet Med. (1998) 15:539–53. doi: 10.1002/(SICI)1096-9136(199807)15:7<539::AID-DIA668>3.0.CO;2-S, 9686693

[15] Kinyoun J Barton F Fisher M Hubbard L Aiello L Ferris F 3rd. Detection of diabetic macular edema. Ophthalmoscopy versus photography--early treatment diabetic retinopathy study report number 5. The ETDRS research group. Ophthalmology. (1989) 96:746–50. doi: 10.1016/s0161-6420(89)32814-4 2740076

[16] American Diabetes Association Professional Practice Committee. 2. Diagnosis and classification of diabetes: standards of care in diabetes-2024. Diabetes Care. (2024) 47:S20–42. doi: 10.2337/dc24-S002 38078589 PMC10725812

[17] Wilkinson CP Ferris FL 3rd Klein RE Lee PP Agardh CD Davis M . Proposed international clinical diabetic retinopathy and diabetic macular edema disease severity scales. Ophthalmology. (2003) 110:1677–82. doi: 10.1016/S0161-6420(03)00475-5, 13129861

[18] Zhou H Wang J Cui X. Causal effect of immune cells, metabolites, cathepsins, and vitamin therapy in diabetic retinopathy: a mendelian randomization and cross-sectional study. Front Immunol. (2024) 15:1443236. doi: 10.3389/fimmu.2024.1443236, 39430744 PMC11487118

[19] Paez-Hurtado AM Calderon-Ospina CA Nava-Mesa MO. Mechanisms of action of vitamin B1 (thiamine), B6 (pyridoxine), and B12 (cobalamin) in pain: a narrative review. Nutr Neurosci. (2023) 26:235–53. doi: 10.1080/1028415X.2022.2034242, 35156556

[20] Deshmukh SV Prabhakar B Kulkarni YA. Water soluble vitamins and their role in diabetes and its complications. Curr Diabetes Rev. (2020) 16:649–56. doi: 10.2174/1573399815666190916114040, 31526351

[21] Rashid R Sohrabi C Kerwan A Franchi T Mathew G Nicola M . The STROCSS 2024 guideline: strengthening the reporting of cohort, cross-sectional, and case-control studies in surgery. Int J Surg. (2024) 110:3151–65. doi: 10.1097/JS9.0000000000001268, 38445501 PMC11175759

